# Supplementary material for: Robust Assessment of Macromolecular Fraction (MMF) in Muscle with Differing Fat Fraction Using Ultrashort Echo Time (UTE) Magnetization Transfer Modeling with Measured T1
Source: Diagnostics (Basel). 2023 Feb 24;13(5):876. doi: 10.3390/diagnostics13050876 (PMC10001337; doi:10.3390/diagnostics13050876)
Supplement: Supplementary file 1 [file diagnostics-13-00876-s001.zip › diagnostics-2186709-supplementary.pdf]

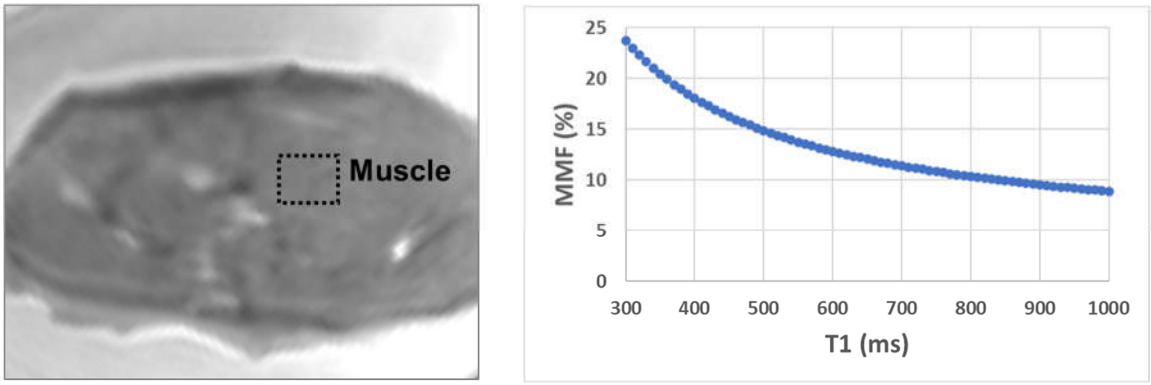


**(A)** **(B)**

**Figure S1.** (**A**) T1-weighted UTE MRI image of a bovine muscle specimen embedded in lard. (**B**) The MMF values of the muscle region (ROI inside of a black box in (**A**)) using differing T1 values as inputs for two-pool MT modeling. T1 value reduction from 1000 ms to 300 ms resulted in over 100% MMF overestimation for the same ROI in pure muscle.
